# Neurocognitive consequences of iatrogenicphthalate exposure during critical illness in children: a study of a development and validation cohort

**DOI:** 10.1186/2197-425X-3-S1-A850

**Published:** 2015-10-01

**Authors:** S Verstraete, I Vanhorebeek, A Covaci, F Güiza, G Malarvannan, PG Jorens, G Van den Berghe

**Affiliations:** KU Leuven, Laboratory of Intensive Care Medicine, Leuven, Belgium; University of Antwerp, Toxicology Center, Antwerp, Belgium; Intensive Care Medicine, University of Antwerp, Antwerp, Belgium

## Introduction

Phthalates are chemical additives incorporated in PVC-based materials to make them more pliable. Intensive care management, especially in the pediatric setting, relies heavily on the use of soft and flexible indwelling medical devices. As phthalates are not chemically bound to these devices, they can leach during use [1,2]. Environmental phthalate exposure has been associated with attention deficit disorders in children [3,4].

## Objectives

We hypothesized that in children treated in the pediatric intensive care unit (PICU), phthalates leaching from the indwelling medical devices contribute to the important long-term attention deficit.

## Methods

Plasma concentrations of di(2-ethylhexyl)phthalate (DEHP) metabolites were quantified in 100 healthy children and in 449 children who had been treated in PICU and were neurocognitively tested 4 years later. In a development patient cohort (N = 228), a multivariable bootstrap study identified the stable thresholds of the exposure to DEHP metabolites that appeared toxic for neurocognitive development. Subsequently, in a second patient cohort (N = 221), the observed independent associations were validated.

## Results

Mean plasma concentrations of the DEHP metabolites were 45- to 280-times higher than normal upon PICU admission, decreased rapidly but remained 3- to 29-times elevated until PICU discharge (all P < 0.001). After adjusting for baseline risk factors and for duration of PICU stay, and further for complications occurring during PICU stay, exceeding the toxic threshold for exposure to the total DEHP metabolites was independently associated with the attention deficit (all P≤0.01) and with the impaired motor coordination (all P≤0.04). The association between phthalate exposure and the attention deficit was confirmed in the validation cohort (all P≤0.01). The size of these effects was the equivalent of 34%-68% of the differences between patients and controls.

## Conclusions

Iatrogenic exposure to DEHP metabolites during intensive care was independently and robustly associated with the important attention deficit observed in children 4 years after pediatric critical illness.

Trial registration at ClinicalTrials.gov: NCT00214916

## Grant Acknowledgment

This work was supported by the Research Foundation-Flanders (FWO), Belgium (FWO fellowship to S. Verstraete); by the Methusalem program of the Flemish government (through the University of Leuven to G. Van den Berghe, METH/08/07 and to G. Van den Berghe and I. Vanhorebeek, METH14/06); by an ERC Advanced Grant (AdvG-2012-321670) from the Ideas Program of the European Union 7th framework program to G. Van den Berghe; and by the Institute for Science and Technology, Flanders, Belgium (through the University of Leuven to G. Van den Berghe, IWT/070695/TBM).
